# Magnitude and determinants of gestational weight gain in Ethiopia: a systematic review and meta-analysis

**DOI:** 10.1186/s40748-026-00270-x

**Published:** 2026-06-10

**Authors:** Getachew Tilaye Mihiret, Yilkal Dagnaw Melesse, Abebaw Abeje Mulueh, Aysheshim Asnake Abneh, Atsede Alle Ewunetie, Haile Amha, Anteneh Lamesgen, Getnet Gedif, Asmamaw Getnet, Alehegn Aderaw Alamneh, Tadele Derbew Kassie, Melkamu Siferih, Menberu Gete

**Affiliations:** 1 Department of Midwifery, College of Medicine and Health Sciences, Debre Markos University, Debre-Markos, Ethiopia; 2 Department of Public Health, College of Medicine and Health Sciences, Debre Markos University, Debre-Markos, Ethiopia; 3 Department of Nursing, College of Medicine and Health Sciences, Debre Markos University, Debre-Markos, Ethiopia; 4 Department of Human Nutrition, College of Medicine and Health Sciences, Debre Markos University, Debre-Markos, Ethiopia; 5 Department of Obstetrics and Gynecology, College of Medicine and Health Sciences, Debre Markos University, Debre-Markos, Ethiopia

**Keywords:** Gestational weight gain, Determinants, Pregnancy, Antenatal care, Ethiopia

## Abstract

**Background:**

Gestational weight gain (GWG) is a key determinant of maternal and neonatal health and is strongly associated with pregnancy outcomes as well as long-term risks, including obesity and metabolic disorders. There is conflicting evidence about the magnitude and determinants of GWG in Ethiopia, no comprehensive synthesis has been conducted to summarize the overall prevalence and associated factors of GWG.

**Objectives:**

Estimate pooled prevalence of GWG categories (inadequate, adequate, and excessive) and identify determinants among pregnant women in Ethiopia.

**Methods:**

A systematic search was conducted in PubMed, Web of Science, African Index Medicus, Sciences Direct, and Google Scholar up to October 2025. Study quality was assessed via the Newcastle–Ottawa Scale. Pooled estimates were calculated via a random-effects model. Statistical heterogeneity was assessed via the I² statistic, and publication bias was evaluated using funnel plots and Egger’s test.

**Results:**

Among the 13 included studies (*n* = 6,099), the pooled mean GWG was 9.93 kg (95% CI: 8.74–11.12). The pooled prevalence of adequate GWG was 41.3% (95% CI: 30.2–52.5), whereas inadequate GWG was highly prevalent at 52.8% (95% CI: 39.8–65.8). The prevalence of excessive GWG was relatively low 7.7% (95% CI: 4.6–10.9). Factors such as frequent meal intake ( > = 3/day) (OR = 2.92, 95% CI: 1.32–4.52), consumption of animal-source foods (OR = 2.70, 95% CI: 1.21–4.19), and > = 3 antenatal care visits (OR = 3.15, 95% CI: 1.47–4.82) were positively associated with adequate GWG. Pre-pregnancy underweight was a significant risk factor for inadequate GWG (OR = 3.31, 95% CI: 1.45–5.18), whereas urban residence was associated with an increased risk of excessive GWG (OR = 5.22, 95% CI: 2.43–11.22).

**Conclusion:**

Ethiopia faces a dual burden of high inadequate GWG prevalence and emerging excessive GWG risk. Integrating tailored nutritional counseling and routine GWG monitoring into antenatal services, alongside context-adapted guidelines, is recommended for improving maternal and fetal health outcomes.

**Clinical trial number:**

Not applicable.

**Supplementary Information:**

The online version contains supplementary material available at 10.1186/s40748-026-00270-x.

## Introduction

Gestational weight gain (GWG) is a critical component of maternal health, influencing both short- and long-term outcomes for mothers and their offspring. Adequate GWG reduces risks of preterm birth, low birth weight, and maternal complications, while inadequate or excessive GWG has been linked to intrauterine growth restriction, macrosomia, gestational diabetes, hypertensive disorders, and postpartum obesity [1, 2]. The Institute of Medicine (IOM) guidelines, revised in 2009, provide internationally recognized recommendations for GWG based on pre-pregnancy body mass index (BMI) categories [3, 4]. However, adherence to these guidelines varies widely across populations, particularly in low- and middle-income countries (LMICs), where nutritional challenges and healthcare disparities are prevalent [5].

The determinants of GWG are multi-factorial. Maternal age, parity, pre-pregnancy BMI, dietary diversity, antenatal exercise, psycho-social stress, and health care (ANC) utilization have consistently emerged as predictors [6–8]. In high-income countries, excessive GWG is more common, and it is driven by sedentary lifestyles and high-calorie intake [9]. Conversely, in LMICs, including sub-Saharan Africa, inadequate GWG predominates due to under-nutrition, food insecurity, and limited access to quality ANC services [10].

In Ethiopia, despite there is a good progress in maternal health indicators, under-nutrition remains a major public health challenge, with 32% of pregnant women being undernourished [11]. Dietary practices are influenced by sociocultural norms, seasonal food availability, and economic constraints [12]. Moreover, despite improvements in ANC coverage, significant gaps remain in the quality of services and the provision of maternal nutrition counseling [13]. These factors collectively contribute to sub-optimal GWG patterns among pregnant women in Ethiopian, with implications for maternal and neonatal outcomes [14].

Gestational weight gain is a critical determinant of maternal and neonatal health, and it influenced by complex biological, behavioral, and socioeconomic factors [15]. In Ethiopia existing evidence remains fragmented, with substantial variation across regions, study designs, and measurement approaches. A systematic synthesis is therefore needed to generate a pooled prevalence and associated determinants. This systematic review and meta-analysis, conducted in accordance with the Preferred Reporting Items for Systematic Reviews and Meta-Analyses (PRISMA) guidelines, aims to synthesize available evidence on GWG and its determinants among pregnant Ethiopian women. The findings will support national and global efforts to improve maternal nutrition, optimize pregnancy outcomes, enhance ANC counseling, and guide effective interventions.

## Methods and materials

### Reporting protocols

This review was reported in accordance with the 2020 PRISMA guidelines [16].

### Eligibility criteria

This systematic review included observational studies that reported quantitative data on the prevalence and associated factors of adequate, inadequate, or excessive GWG among pregnant women. Articles that were not fully accessible were excluded, as the absence of full texts prevented adequate assessment of methodological quality.

### Searching strategy

A comprehensive systematic search was performed across major international databases including PubMed, Web of Science, African Index Medicus (AIM), and Sciences Direct from October 10 to October 30, 2025. Additional searches were conducted in Google Scholar and through manual screening to identify unpublished studies and gray literature.

The PubMed search strategy used combinations of keywords and Boolean operators, including: “weight gain” OR “gestational weight gain” OR “adequate weight gain” OR “inadequate weight gain” OR “excessive weight gain” AND “pregnant women” OR “women” OR “woman” AND “Ethiopia” OR “Democratic Republic of Ethiopia.” No restrictions were applied on publication date. Reference lists of eligible articles were also hand-searched to identify additional relevant studies.

### Study selection procedure

This review included all published and gray literature in English that reported the magnitude of GWG (adequate, inadequate, or excessive) or examined its determinants or associated factors. After removing duplicates via EndNote X7, two reviewers (GTM and YDM) independently screened titles, abstracts, and full texts to determine study eligibility. Discrepancies were resolved through discussion, with a third reviewer (AAM) consulted when consensus could not be reached. Articles that were not fully accessible or did not report the outcomes of interest were excluded. The full texts of the studies that passed the initial screening were reviewed and in cases of duplicate data, the most complete or most recent publication was retained.

### Data extraction

Two reviewers (GTM and MG) independently extracted data via a standardized Microsoft™ word extraction tool adapted from the Joanna Briggs Institute (JBI) [17]. A third author (AAA) cross‑checked the extracted information and resolved discrepancies through discussion and consensus. The extracted data included study characteristics (author, year, setting, and sample size), study design, participant characteristics, and the magnitude of GWG. For the second outcome, information on determinants or associated factors of weight gain during pregnancy was collected, and log odds ratios for each factor were calculated on the basis of the original study findings.

### Quality and bias assessment

The risk of bias of the included articles was independently assessed by two authors (HA and AG) using the tool adapted from Hoy et al. [18]. This instrument comprises ten items covering the four domains of bias, along with an overall risk-of-bias judgment. Any disagreements between reviewers were resolved through discussion with a third reviewer (MS). Each item was rated as either low or high risk, with “unclear” responses classified as high risk. Based on the number of high-risk items, studies were categorized as having low (≤ 2), moderate ([3–4]), or high (≥ 5) risk of bias. Quality assessment was also independently conducted by two reviewers (GTM and AAE). Their appraisal scores were compared, and discrepancies were resolved by a third reviewer (GG) before final scoring. Study quality was evaluated using the Newcastle–Ottawa Scale (NOS) [19]. The scale includes three components: assessment of outcomes and statistical analysis (maximum of two stars), evaluation of methodological quality (up to five stars), and comparability of study groups or cohorts (up to two stars). Studies scoring ≥ 5 out of 10 stars were classified as high quality.

### Outcome variables

#### Early pregnancy weight

maternal weight measured before 16 weeks of gestation [14].

#### Last pregnancy weight

The last weight recorded immediately before delivery [14].

#### Gestational weight gain

The difference between the last pregnancy weight and the early pregnancy weight [6].

The Institute of Medicine guidelines, revised in 2009, provide internationally recognized recommendations for GWG based on BMI. According to these guidelines, GWG is classified as inadequate, adequate, or excessive [3, 4]. The recommended total weight gain ranges are as follows:

- **Underweight (BMI < 18.5 kg/m²)**: 12.5–18 kg.
- **Normal weight (BMI 18.5–24.9 kg/m²)**: 11.5–16 kg.
- **Overweight (BMI 25.0–29.9 kg/m²)**: 7–11.5 kg.
- **Obesity (BMI ≥ 30.0 kg/m²)**: 5–9 kg [20].

### Measure of outcome

We conducted a meta-analysis and reported pooled estimates as odd ratio (ORs) with 95% confidence interval (CIs) for dichotomous data. The 95% CI prevalence was calculated by the formula: prevalence ± standard error and the standard error for each original study were calculated via the binomial distribution formula: (prevalence*(100-prevalence)/sample size).

### Assessment of heterogeneity

Heterogeneity among reported prevalence estimates was assessed by inspecting forest plots for overlap of confidence intervals and by calculating Higgins’s I² statistic. The I² statistic quantifies the percentage of variation across studies that are due to heterogeneity rather than sampling error, and it ranges from 0% to 100%. An I² value of 0% indicates statistical homogeneity. Values of 25%, 50%, and 75% are interpreted as representing low, moderate, and substantial heterogeneity, respectively [21].

### Strategy for data synthesis

The data were analyzed using STATA/SE version 17. A random-effects meta-analysis model was applied to pool prevalence estimates and odds ratios (ORs) with corresponding 95% confidence intervals (CIs). Statistical pooling was conducted separately for each outcome of interest. Heterogeneity across studies was assessed using Higgins’ I² statistic, with values < 49%, 50–75%, and > 75% indicating low, moderate, and high heterogeneity, respectively. Because substantial heterogeneity was observed, all pooled estimates were generated using a random-effects model, which accounts for both within- and between-study variability. Subgroup and sensitivity analyses were performed to explore potential sources of heterogeneity. Publication bias was evaluated visually using funnel plots and statistically using Egger’s regression test at the 5% significance level. In the forest plots, the size of each box reflects the weight assigned to each study, and the horizontal lines represent the 95% CI.

## Results

### Study selection process

**Fig. 1:**
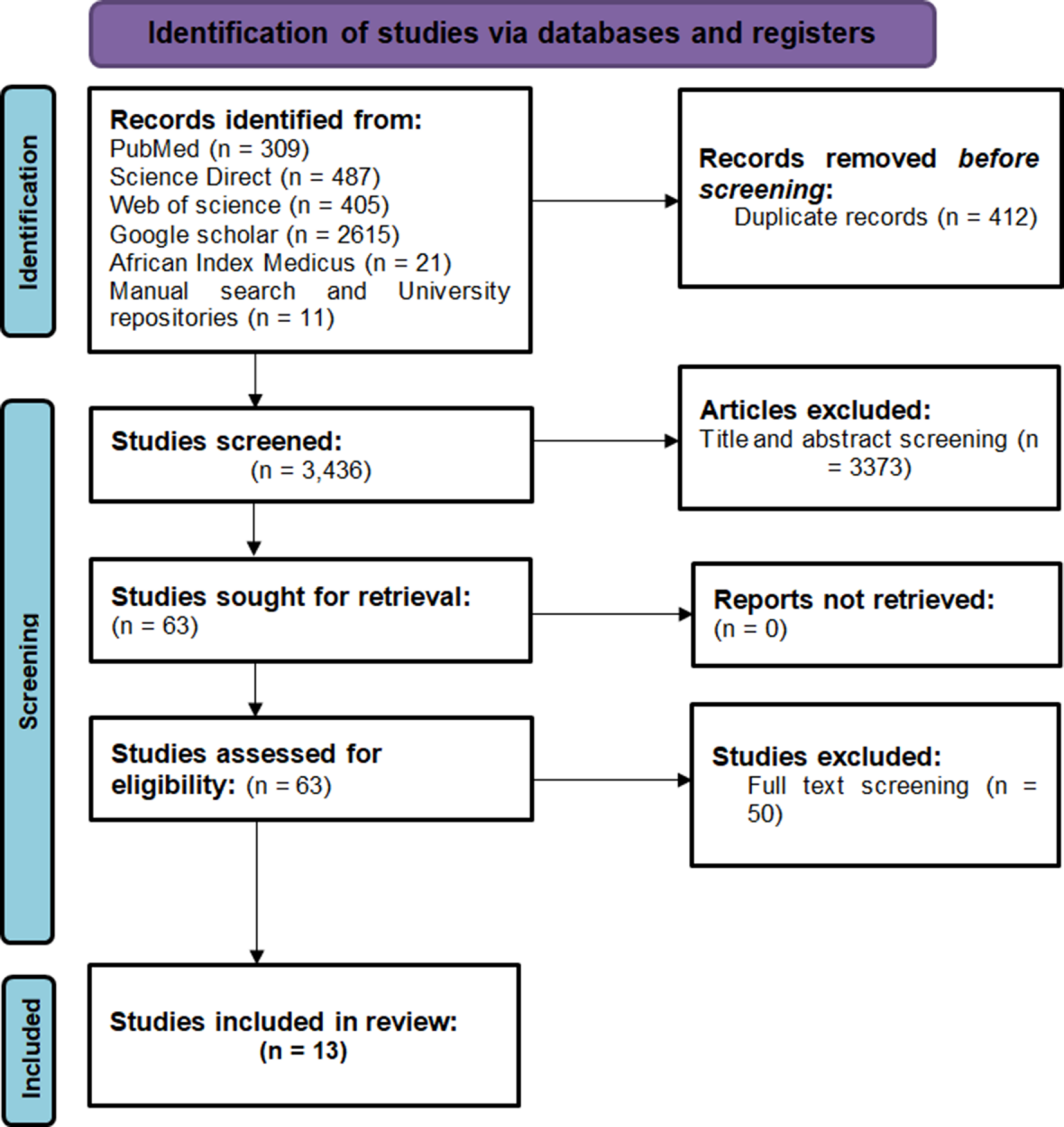
The selection process for studies on gestational weight gain in Ethiopia

A total of 3,848 articles were retrieved from MEDLINE (PubMed), African Index Medicus (AIM), ScienceDirect, Web of Science, Google Scholar, manual searches, and university repositories. After removing 3,373 records that were ineligible based on title and abstract screening, and 412 duplicates, 63 full-text articles were assessed for eligibility among these studies, 13 met the inclusion criteria and were included in the final meta-analysis (Fig. 1).

### Characteristics of included studies

A total of 13 studies published between 2016 and 2025 were included in the final meta-analysis, encompassing 6,099 pregnant women across multiple Ethiopian regions [6, 7, 14, 15, 22–30]. Study designs ranged from cohort (*n* = 6) and cross-sectional (*n* = 5) to one RCT and one case–control study. Sample sizes varied from 223 to 934 participants. Nearly all studies used the IOM 2009 guidelines to define GWG categories, except for one study, which applied the Ethiopian National Antenatal Guideline [15] (Table 1).

**Table 1:** Characteristics of included studies (*n* = 13)

Author (Year)	Study Setting	Study Design	Sample Size	Mean GWG (kg)	Adequate GWG (%)	Inadequate GWG (%)	Excessive GWG (%)	Quality assessment score
Alemu, M. (2024)	Addis Ababa	Cross-sectional	422	—	88.9	2.8	8.3	7
Alemu, M., et al. (2025)	Amhara	Cross-sectional	517	8.4	30.9	60.1	—	6
Asefa, F., et al. (2016)	Harari Region	Cross-sectional	411	8.96	28.0	69.3	2.7	8
Asefa, F., et al. (2021)	Addis Ababa	Prospective cohort	395	8.7	27.9	67.2	4.9	8
Beressa, G., et al. (2025)	Oromia	RCT	223	10.5	30.5	65.9	3.6	8
Beyene, G. et al. (2024)	SNNPR	Prospective cohort	424	13.3	56.0	26.0	18.0	8
Chaltu, F. (2022)	Oromia Region	Cross-sectional	336	8.38	25.3	69.6	—	7
Derese Asfaw, T., et al. (2025)	Dire Dawa City	Case-Control	438	—	47.0	53.0	—	8
Engidaw, M. T., et al. (2023)	Amhara Region	Prospective cohort	422	—	65.1	21.5	13.4	9
Hawulte, M., et al. (2023)	Oromia Region	Cross-sectional	360	—	34.2	65.8	—	7
Misgina, K. H., et al. (2021)	Tigray Region	Prospective cohort	934	10.6	36.0	64.0	—	9
Tela, F. G., et al. (2019)	Tigray Region	Prospective cohort	332	12.0	—	—	—	8
Terfassa, T. G., et al. (2025)	Oromia Region	Prospective cohort	885	—	26.0	69.0	5.0	8

Note: ***GWG***: *Gestational weight gain; “—” indicates data not reported*

### Risk of bias

A comprehensive risk of bias assessment was performed for all included studies using a standardized ten-item tool adapted from Hoy et al. [18]. The evaluations indicated a low risk of bias for 12 of the 13 included studies, with the remaining one study [15] presenting a high risk.

## Meta-analysis

### Pooled magnitude of gestational weight gain

The pooled mean GWG was 9.93 kg (95% CI: 8.74–11.12), lower than IOM recommendations, with low-to-moderate heterogeneity (I² = 29.2%). We used a random-effects model a priori (before seeing the data) due to anticipated methodological heterogeneity across studies, even when statistical heterogeneity was low (Supplementary: Fig. 1).

### Pooled prevalence of GWG categories

**Fig. 2:**
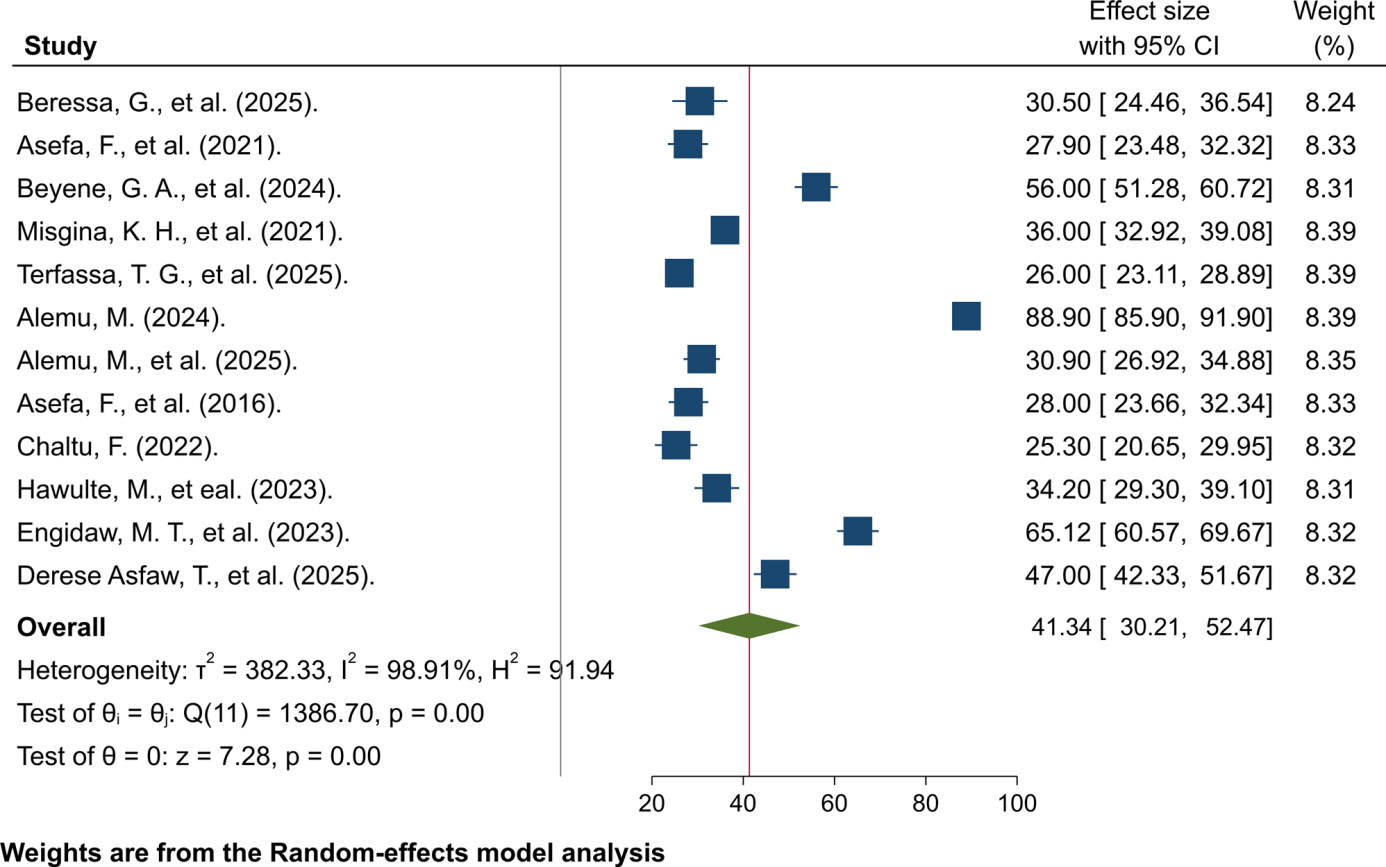
Pooled estimate of adequate gestational weight gain in Ethiopia

**Adequate GWG**: 41.3% (95% CI: 30.2–52.5) **(**Fig. 2**).**

**Fig. 3:**
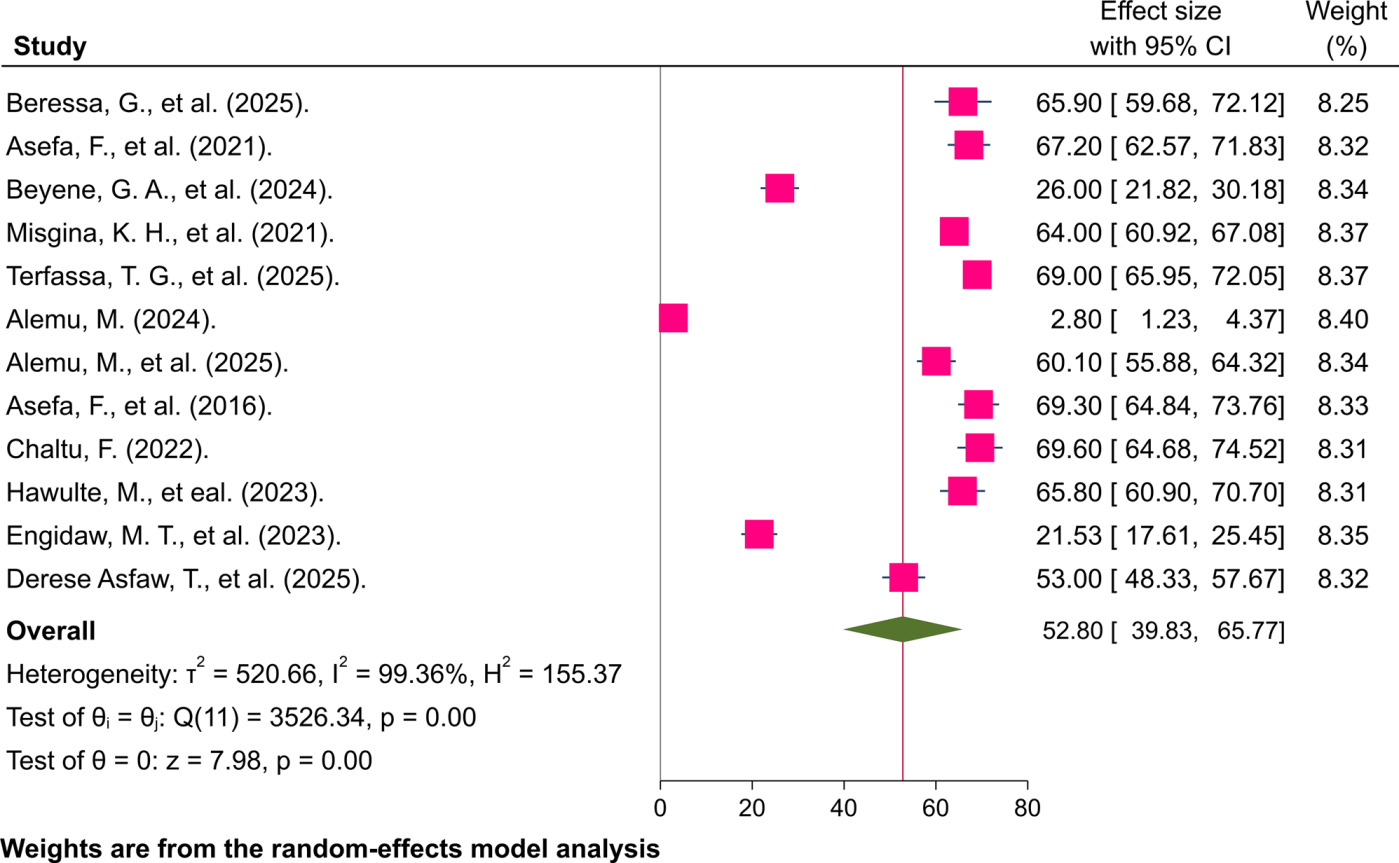
Pooled estimate of insufficient gestational weight gain in Ethiopia

**Inadequate GWG**: 52.8% (95% CI: 39.8–65.8) (Fig. 3).

**Fig. 4:**
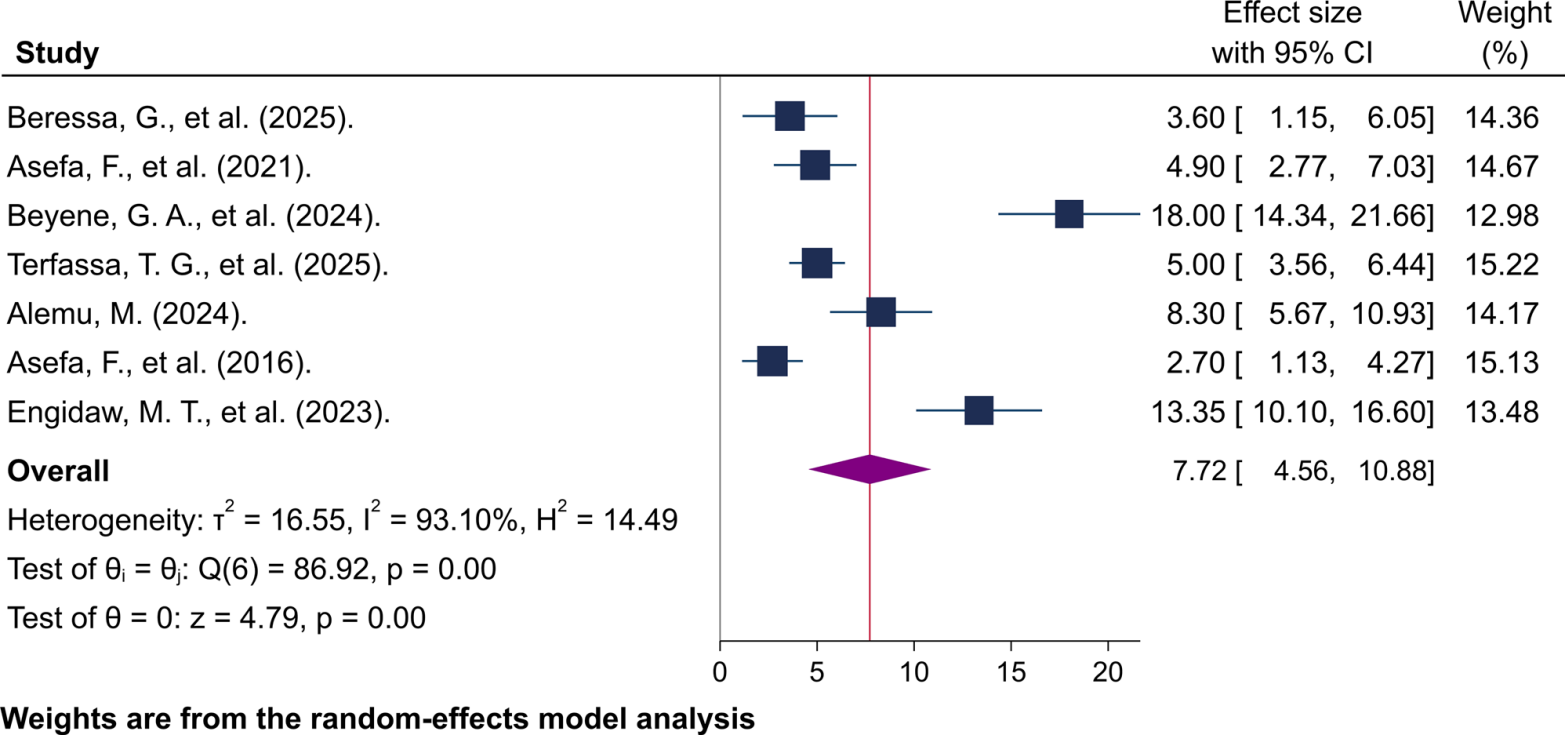
Pooled estimate of excessive gestational weight gain in Ethiopia

**Excessive GWG**: 7.7% (95% CI: 4.6–10.9) (Fig. 4).

Inadequate GWG was the most common deviation, affecting more than half of women. All categorical outcomes showed high heterogeneity (I² > 90%), likely may be due to differences in study design, region, and year.

### Subgroup analysis

Substantial heterogeneity was observed in the pooled prevalence estimates of adequate GWG, inadequate GWG, and excessive GWG, with I² values exceeding 90%, indicating considerable variability across the included studies. Subgroup analyses were conducted for each category of GWG by study design, geographic region, and study year revealed variability but did not fully explain heterogeneity (Supplementary: Figs.2, 3 and 4). 

### Sensitivity analysis

**Fig. 5:**
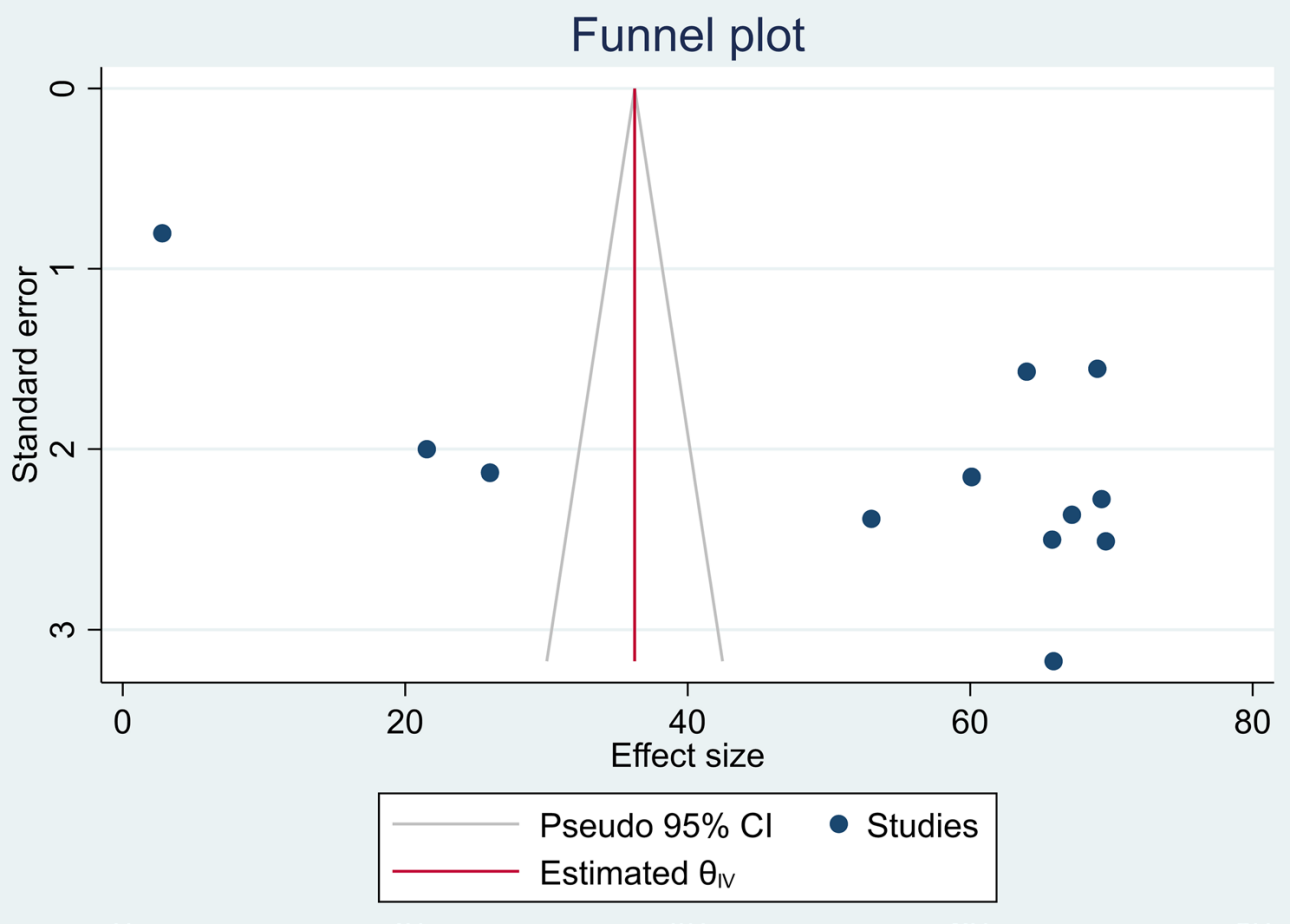
Meta funnels presentations of the proportion of adequate gestational weight gain

**Fig. 6:**
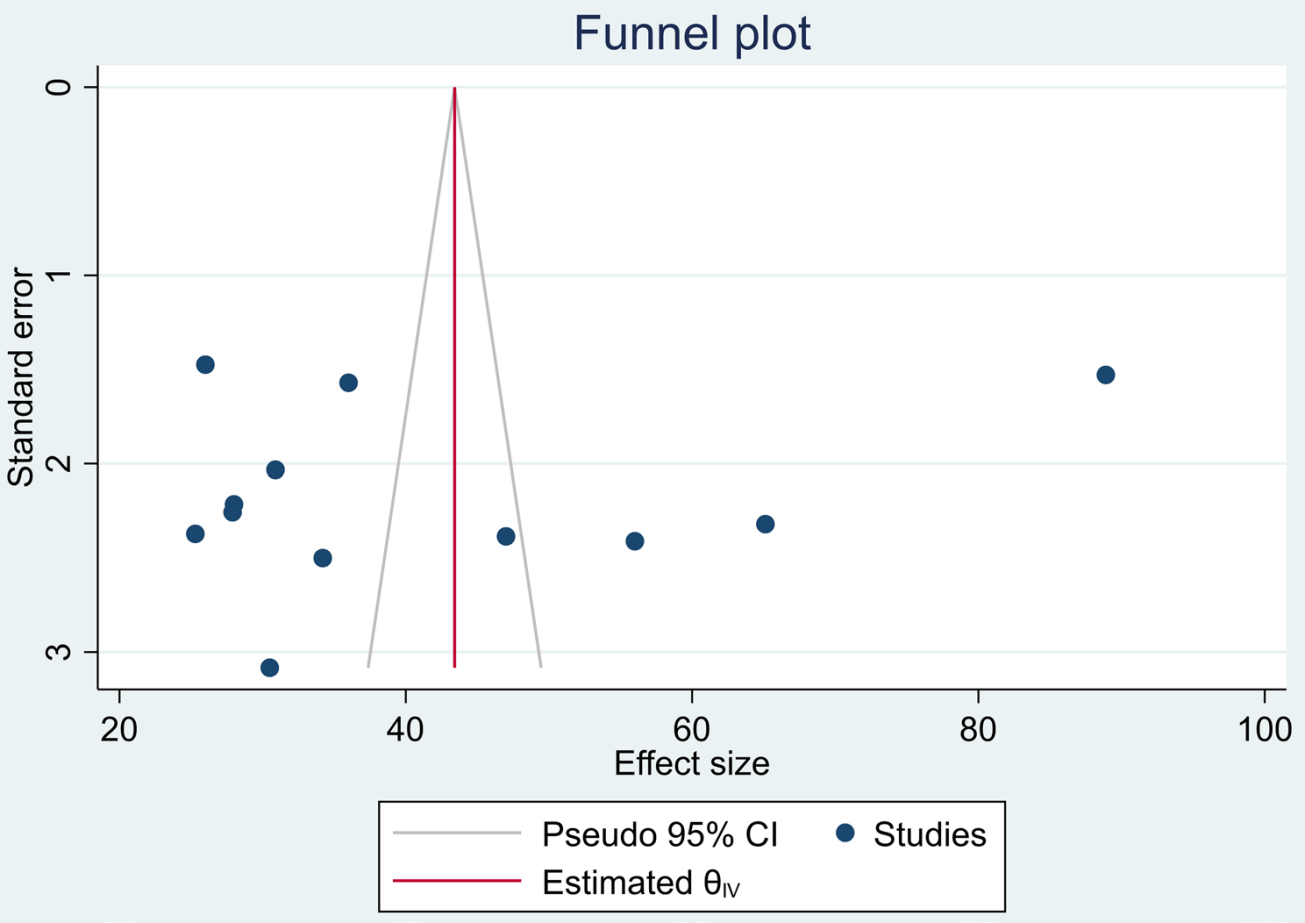
Meta funnels presentations of the proportion of inadequate gestational weight gain

**Fig. 7:**
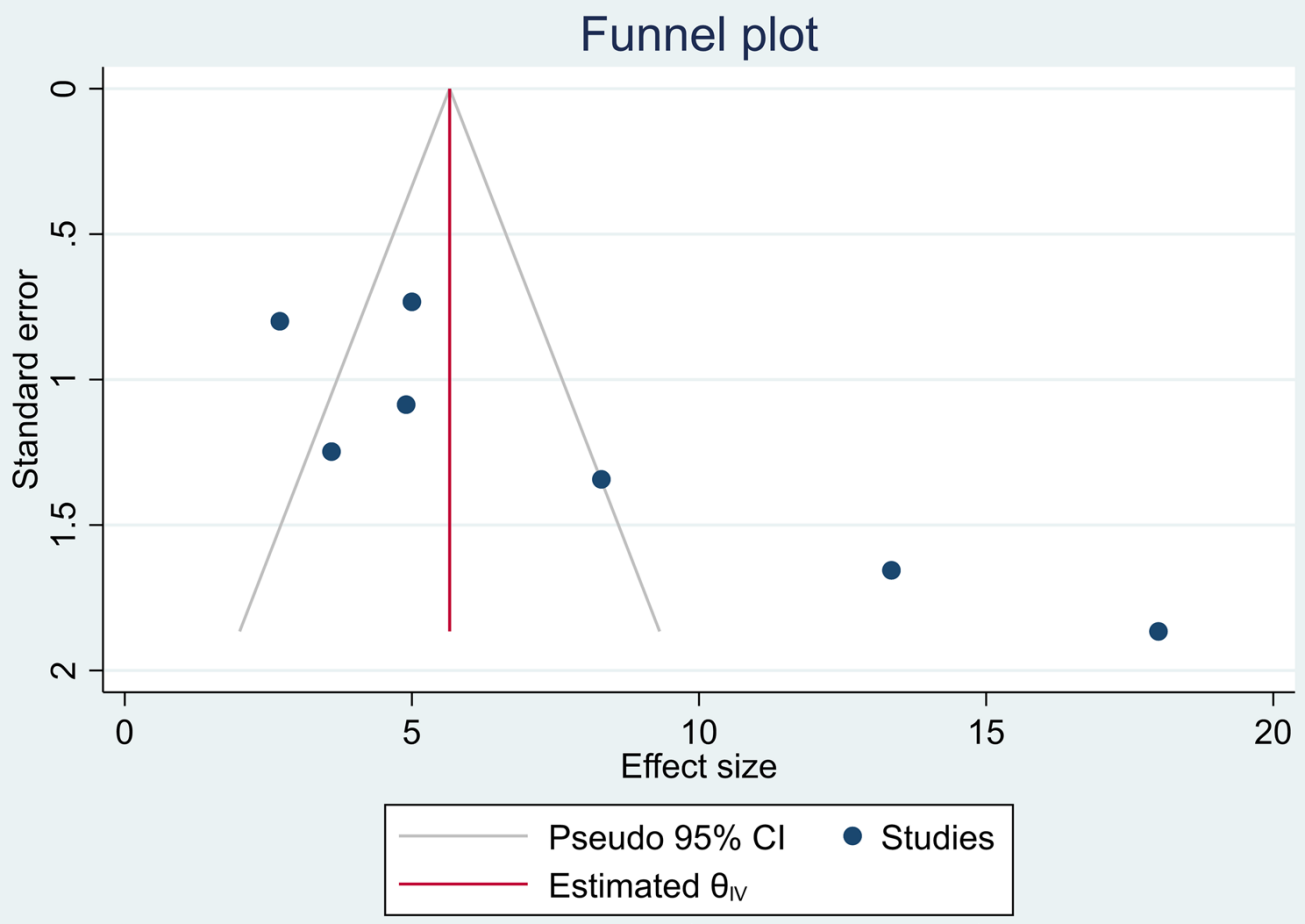
Meta funnels presentations of the proportion of excessive gestational weight gain

Sensitivity analysis was conducted separately for each GWG outcome. Sensitivity analyses confirmed that no single study disproportionately influenced pooled estimates. These detailed plots are provided in supplementary materials (Supplementary: Figures 5, 6 and 7).

### Meta-regression analysis

Study year and sample size were not significant predictors of heterogeneity across GWG categories, suggesting other contextual factors drive variability. For excessive GWG, study year was not significantly associated with the pooled prevalence estimate (coefficient = 0.581; 95% CI: -0.771-1.932; *p* = 0.400). Likewise, sample size did not significantly explain between-study heterogeneity (coefficient = -0.005; 95% CI: -0.029–0.018; *p* = 0.654). Similarly, sample size was not a significant predictor of heterogeneity for adequate GWG (coefficient = − 0.013; 95% CI: -0.078–0.053; *p* = 0.704) or inadequate GWG (coefficient = 0.017; 95% CI: -0.071–0.105; *p* = 0.704).

The persistence of substantial heterogeneity, despite subgroup and meta-regression analyses, indicates the potential influence of unmeasured or inadequately reported factors. These may include variations in maternal nutritional status prior to pregnancy, disparities in household and regional food security, and differences in the quality and coverage of ANC services. Future primary studies should aim to consistently report these variables to enable more comprehensive meta-analytic assessments.

### Assessment of publication bias

Funnel plots indicated no bias for adequate GWG **(**Fig. 5**).** Consistent with this finding, Egger’s intercept test showed no evidence of small-study effects (intercept = − 10.08; 95% CI: −35.49–15.32; *p* = 0.44), indicating no significant publication bias.

In contrast, potential small-study effects were observed for both inadequate and excessive GWG. Visual inspection of funnel plots suggested asymmetry (Figs. 6 and 7), which was supported by Egger’s test for inadequate GWG (intercept = 21.84; 95% CI: 2.33 to 40.35; *p* = 0.028) and excessive GWG (intercept = 11.63; 95% CI: 6.18 to 17.08; *p* < 0.001). However, the trim-and-fill analysis did not impute any additional studies, and the pooled effect estimates remained unchanged after adjustment (inadequate GWG: 52.8% [95% CI: 39.8–65.8]; excessive GWG: 7.7% [95% CI: 4.6–10.9]). These findings suggest that, although statistical evidence of small-study effects was observed, their impact on the overall pooled estimates is likely minimal.

On the other hand, these findings should be interpreted with caution, particularly in the context of LMICs such as Ethiopia, where publication bias may arise from the underrepresentation of small-scale or rural studies with null findings, limited dissemination of non-significant results, and reliance on facility-based data. Such factors may contribute to the observed asymmetry despite the stability of pooled estimates.

### Determinants of gestational weight gain

This study synthesized the factors influencing GWG into three main themes:

### • Nutritional/dietary

- Dietary behaviors emerged as key modifiable determinants of GWG. Frequent meals (> 3/day) and daily animal-source food intake were positively associated with adequate GWG (pooled OR ~ 2.7–2.9).

#### • Early-pregnancy BMI

- Underweight women had over threefold higher odds of inadequate GWG (OR = 3.31), while overweight/obese women were more likely to experience higher GWG categories.

#### • Healthcare/socioeconomic

- More than three > 3 ANC visits increased odds of adequate GWG (pooled OR = 3.15). Additionally, a single study reported that urban residence was associated with excessive GWG (AOR = 5.22, 95% CI: 2.43–11.22) [25].
- Associations between education and employment were complex and outcome specific. Higher education was generally protective against adequate GWG, while its relationship with inadequate GWG was inconsistent (Table 2).

**Table 2:** The pooled determinants of gestational weight gain

Determinant Category	Author(s)	Factors	Outcome Variable	Pooled odds ratio	95% CI	I² (%)
Dietary Factors	Alemu, M., et al. (2025) [15]. Chaltu. F. (2022)[27].	Meal frequency > 3 times/day	Adequate GWG	2.92	1.32–4.52	0.00
	Alemu, M., et al. (2025) [15]. Asefa, F., et al. (2016) [29].	Daily animal-source food consumption	Adequate GWG	2.70	1.21–4.19	0.00
	Hawulte, M., et eal. (2023) [24]. Asefa, F., et al. (2021) [6].	Low dietary diversity (< 5 food groups)	Inadequate GWG	2.38	0.39–14.44	0.35
Early-pregnancy BMI	Hawulte, M., et eal. (2023) [24]. Asefa, F., et al. (2021) [6].	Underweight	Inadequate GWG	3.31	1.45–5.18	0.00
Healthcare Utilization	Alemu, M., et al. (2025) [15]. Asefa, F., et al. (2016) [29].	ANC visits > 3	Adequate GWG	3.15	1.47–4.82	0.00

Note: heterogeneity assessed using the I² statistic

## Discussion

This systematic and meta-analysis provides the first nationally pooled estimates of GWG categories and determinants in Ethiopia. The pooled mean GWG was found to be 9.93 kg which is substantially lower than the GWG range recommended by the IOM, particularly for women with normal and underweight pre-pregnancy BMI categories [3, 31].

Inadequate GWG affects more than half of pregnant women (52.8%), which is comparable to reports from Nigeria 62.9 [32], Bangladesh 54% [33], Nepal 43.3% [34], and a regional meta-analysis from sub-Saharan Africa [35]. A recent Ethiopian cohort study similarly found that approximately 69% of pregnant women experienced insufficient weight gain, suggesting that under-nutrition remains a major public health challenge, with 32% of pregnant women being undernourished [11]. This finding may underscore a persistent burden of maternal under-nutrition during pregnancy and reflects broader nutritional challenges faced by women in low-income settings [36].

This review revealed that excessive GWG remains relatively low (7.7%) but is emerging in urban populations. In contrast, studies from Europe, North America, and China report excessive GWG prevalence exceeding 40% [37, 38]. The relatively low prevalence of excessive observed in Ethiopia likely reflects the country’s current stage of nutritional transition. However, emerging evidence suggests that urbanization may accelerate the shift toward excess weight gain in specific subpopulations [7, 25]. Notably, increased consumption of refined cereals and vegetable oil a key feature of the nutrition transition has been documented in urban Ethiopia [39].

Dietary behaviors are key modifiable determinants of GWG. Frequent meal intake (> 3 meals/day) and daily consumption of animal-source foods were consistently associated with adequate GWG. These findings supported by previous evidences from studies in Sweden [40], India [41] and USA [42] which demonstrated that higher meal frequency and dietary protein adequacy improve gestational weight trajectories and fetal growth. In contrast, in this study, the pooled analysis did not reveal a statistically significant association between low dietary diversity and inadequate GWG. This inconsistency may be explained by differences in dietary diversity measurement tools, seasonal food availability, and residual confounding. Similar findings have been reported in a systematic review in Brazil suggesting that dietary diversity alone may be an insufficient proxy for caloric adequacy in resource-limited settings [43].

Early-pregnancy BMI was a strong and consistent predictor of GWG outcomes. Underweight women had more than threefold higher odds of inadequate GWG. Maternal under-nutrition prior to conception constrains GWG and increases the risk of adverse birth outcomes [44]. Conversely, overweight and obese women were substantially more likely to experience higher GWG categories, including excessive GWG. These findings are supported by previous studies from Ghana [44], Asia [45], Europe [37], and emphasize the growing burden of malnutrition in Ethiopia. The coexistence of under-nutrition and emerging overweight highlights the urgent need for a life-course approach to maternal nutrition, integrating preconception care, BMI screening, and tailored counseling.

This meta-analysis revealed that antenatal care utilization is a critical protective factor. Women who had more than 3 ANC contact were significantly more likely to achieve adequate GWG, which is consistent with evidence from Brazil [46], and WHO recommendations [47], ANC visits provide opportunities for nutritional counseling, weight monitoring, early detection of complications, and supplementation, all of which contribute to improved GWG outcomes [48]. Urban residence was associated with a markedly increased risk of excessive GWG, reflecting lifestyle transitions, reduced routine physical activity, and increased access to energy-dense foods [49]. Similar urban rural gradients in excessive GWG have been documented in rapidly urbanizing African and Asian settings [41]. Associations with education and employment are complex and outcome specific, reflecting the dual influence of socioeconomic advantage on dietary quality and sedentary behaviors [50].

### Strength and limitation

This review provides the first nationally pooled estimates of GWG and determinants in Ethiopia. The inclusion of a large participant cohort (*N* = 6,099), which exceeds the sample sizes of individual studies, enhances the statistical power of the analysis. However, the high heterogeneity observed for categorical GWG outcomes and reliance on observational studies limit causal inference. Recall bias in pre-pregnancy weight estimation and variability in measurement timing should be considered when interpreting findings. Future research should prioritize longitudinal studies with standardized GWG assessment protocols, deeper investigations into the quality of ANC nutritional counseling, and mixed-methods studies to understand the sociocultural drivers of dietary choices during pregnancy.

Finally, the absence of PROSPERO registration may limit transparency. However, measures such as adherence to PRISMA guidelines, the use of independent reviewers, and searching the PROSPERO database (https://www.crd.york.ac.uk/prospero/) to identify any recently published, completed, or ongoing projects on this topic were applied to minimize bias. No relevant registered or ongoing reviews were found.

## Conclusion

These systematic review and meta-analysis suggest that Ethiopia faces a dual challenge: a persistently high burden of inadequate GWG alongside a rising risk of excessive GWG in urban and higher-BMI populations. Moreover, maternal residency, daily consumption of animal-sourced foods, frequent meal intake (> 3 times per day), having more than 3 ANC and pre-pregnancy BMI were the determinants of GWG during pregnancy. Integrating routine GWG monitoring into ANC services, strengthening nutrition counseling tailored to pre-pregnancy BMI, and promoting diversified, frequent, and protein-rich diets are critical priorities. Context-specific GWG guidelines adapted from IOM recommendations may also be necessary to improve clinical applicability in Ethiopian settings.

## Supplementary Information

Below is the link to the electronic supplementary material.

Supplementary Material 1

Supplementary Material 2

Supplementary Material 3

Supplementary Material 4

## Data Availability

All the data are available in the manuscript.

## Abbreviations

ANC: Antenatal Care
BMI: Body Mass Index
GWG: Gestational Weight Gain
IOM: Institutes of Medicine
LMICs: Low- and Middle-Income Countries
PRISMA: Reporting Items for Systematic Reviews and Meta-Analyses
PROSPERO: International prospective register of systematic reviews
