# Supplementary material for: Magnitude and determinants of gestational weight gain in Ethiopia: a systematic review and meta-analysis
Source: Matern Health Neonatol Perinatol. 2026 Jun 10;12:23. doi: 10.1186/s40748-026-00270-x (PMC13251280; doi:10.1186/s40748-026-00270-x)
Supplement: Supplementary file 1 — Supplementary Material 1 [file 40748_2026_270_MOESM1_ESM.docx]

| **Table S2: Risk of Bias assessment Tool of Eligible Articles** **by using the Hoy 2012 tool** | | | | | | | | | | |  |
| --- | --- | --- | --- | --- | --- | --- | --- | --- | --- | --- | --- |
| **Study ID** | **Representation** | **Sampling** | **Random selection** | **Non response bias** | **Data collection** | **Case Definition** | **Reliability and validity of study tool** | **Method of data collection** | **Prevalence period** | **Numerator and denominator** | **Summary Assessment** |
| Alemu, M. (2024). | Low risk | Low risk | Low risk | Low risk | Low risk | Low risk | Low risk | Low risk | low risk | Low risk | Low risk |
| Alemu, M., et al. (2025). | Low risk | Low risk | Low risk | Low risk | Low risk | Low risk | high risk | Low risk | low risk | high risk | Low risk |
| Asefa, F., et al. (2016). | Low risk | Low risk | Low risk | Low risk | Low risk | Low risk | Low risk | Low risk | Low risk | Low risk | Low risk |
| Asefa, F., et al. (2021). | Low risk | Low risk | Low risk | Low risk | Low risk | Low risk | Low risk | Low risk | Low risk | Low risk | Low risk |
| Beressa, G., et al. (2025). | Low risk | Low risk | Low risk | Low risk | Low risk | Low risk | Low risk | Low risk | Low risk | Low risk | Low risk |
| Beyene, G. A., et al. (2024). | Low risk | Low risk | Low risk | Low risk | Low risk | Low risk | Low risk | Low risk | Low risk | Low risk | Low risk |
| Chaltu, F. (2022). | Low risk | Low risk | Low risk | Low risk | Low risk | Low risk | high risk | Low risk | Low risk | Low risk | Low risk |
| Derese Asfaw, T., et al. (2025). | Low risk | Low risk | Low risk | Low risk | Low risk | Low risk | Low risk | Low risk | low risk | Low risk | Low risk |
| Engidaw, M. T., et al. (2023). | Low risk | Low risk | Low risk | Low risk | Low risk | Low risk | Low risk | Low risk | Low risk | Low risk | Low risk |
| Hawulte, M., et eal. (2023). | Low risk | Low risk | Low risk | Low risk | Low risk | Low risk | high risk | Low risk | low risk | Low risk | Low risk |
| Misgina, K. H., et al. (2021). | Low risk | Low risk | Low risk | Low risk | Low risk | Low risk | Low risk | Low risk | low risk | Low risk | Low risk |
| Tela, F. G., et al. (2019). | Low risk | Low risk | Low risk | Low risk | Low risk | Low risk | Low risk | Low risk | low risk | Low risk | Low risk |
| Terfassa, T. G., et al. (2025). | Low risk | Low risk | Low risk | Low risk | Low risk | Low risk | Low risk | Low risk | low risk | Low risk | Low risk |
| **Risk of bias assessment tool: Yes (low risk); No (high risk):** | | | | | | | | | | |  |
| 1. Representation: Was the study population a close representation of the national population? | | | | | | | | | | |  |
| 2. Sampling: Was the sampling frame a true or close representation of the target population? | | | | | | | | | | |  |
| 3. Random selection: Was some form of random selection used to select the sample OR was a census undertaken? | | | | | | | | | | |  |
| 4. Non-response bias: Was the likelihood of non-response bias minimal? | | | | | | | | | | |  |
| 5. Data collection: Were data collected directly from the subjects? | | | | | | | | | | |  |
| 6. Case definition: Was an acceptable case definition used in the study? | | | | | | | | | | |  |
| 7. Reliability and validity of study tool: Was the study instrument that measured the parameter of interest show to have reliability and validity? | | | | | | | | | | |  |
| 8. Data collection: Was the same mode of data collection used for all subjects? | | | | | | | | | | |  |
| 9. Prevalence period: Was the length of the prevalence period for the parameter of interest appropriate? | | | | | | | | | | |  |
| 10. Numerators and denominators: Were the numerator(s) and denominator(s) for the parameter of interest appropriate? | | | | | | | | | | |  |
| **The overall risk of bias scored based on the number of high risks of bias per study: low risk (≤2), moderate risk (3–4), and high risk (≥5).** | | | | | | | | | | |  |
|  |  |  |  |  |  |  |  |  |  |  |  |
