# Supplementary material for: Magnitude and determinants of gestational weight gain in Ethiopia: a systematic review and meta-analysis
Source: Matern Health Neonatol Perinatol. 2026 Jun 10;12:23. doi: 10.1186/s40748-026-00270-x (PMC13251280; doi:10.1186/s40748-026-00270-x)
Supplement: Supplementary file 2 — Supplementary Material 2 [file 40748_2026_270_MOESM2_ESM.docx]

| **Supplementary 2. Scoring of the quality of articles by authors using the Newcastle-Ottawa Quality Assessment tool.** | | | | | | | | | |
| --- | --- | --- | --- | --- | --- | --- | --- | --- | --- |
|  |  | **Selection** | | | | **Comparability** | **Outcome** | | Total |
| No. | Study | **Presentiveness of the sample** | **Sample size** | **Non-respondents** | **Ascertainment of the exposure (risk factor)** | **Comparability and control cofound** | **Assessment of outcome** | **Statistical test** | **Out of 10** |
| 1 | Alemu, M. (2024). | Truly representative (random sampling) * | Justified and satisfactory. * | Comparable and the response rate is satisfactory. * | Used validated measurement tool. * | The study controls for the most important factor. * | Self-report. * | Clearly described* | 7 |
| 2 | Alemu, M., et al. (2025). | Truly representative (random sampling) * | Justified and satisfactory. * | Comparable and the response rate is satisfactory. * | Used validated measurement tool. ** | The study controls for the most important factor. | Self-report. * | Clearly described | 6 |
| 3 | Asefa, F., et al. (2016). | Truly representative (random sampling) * | Justified and satisfactory. * | Comparable and the response rate is satisfactory. * | Used validated measurement tool. ** | The study controls for the most important factor. * | Self-report. * | Clearly described* | 8 |
| 4 | Asefa, F., et al. (2021). | Truly representative (random sampling) * | Justified and satisfactory. * | Comparable and the response rate is satisfactory. * | Used validated measurement tool. ** | The study controls for the most important factor. * | Self-report. * | Clearly described* | 8 |
| 5 | Beressa, G., et al. (2025). | Truly representative (random sampling) * | Justified and satisfactory. * | Comparable and the response rate is satisfactory. * | Used validated measurement tool. ** | The study controls for the most important factor. * | Self-report. * | Clearly described* | 8 |
| 6 | Beyene, G. A., et al. (2024). | Truly representative (random sampling) * | Justified and satisfactory. * | Comparable and the response rate is satisfactory. * | Used validated measurement tool. ** | The study controls for the most important factor. * | Self-report. * | Clearly described* | 8 |
| 7 | Chaltu, F. (2022). | Truly representative (non-random sampling) * | Justified and satisfactory. * | Comparable and the response rate is satisfactory. * | Used validated measurement tool. * | The study controls for the most important factor. * | Self-report. * | Clearly described* | 7 |
| 8 | Derese Asfaw, T., et al. (2025). | Truly representative (non-random sampling) * | Justified and satisfactory. * | Comparable and the response rate is satisfactory. * | Used validated measurement tool. * | The study controls for the most important factor. ** | Self-report. * | clearly* described | 8 |
| 9 | Engidaw, M. T., et al. (2023). | Truly representative (non-random sampling) * | Justified and satisfactory. * | Comparable and the response rate is satisfactory. * | Used validated measurement tool. ** | The study controls for the most important factor. ** | Self-report. * | clearly* described | 9 |
| 10 | Hawulte, M., et eal. (2023). | Truly representative (non-random sampling) * | Justified and satisfactory. * | Comparable and the response rate is satisfactory. * | Used validated measurement tool. * | The study controls for the most important factor. * | Self-report. * | clearly* described | 7 |
| 11 | Misgina, K. H., et al. (2021). | Truly representative (non-random sampling) * | Justified and satisfactory. * | Comparable and the response rate is satisfactory. * | Used validated measurement tool. ** | The study controls for the most important factor. ** | Self-report. * | Clearly described* | 9 |
| 12 | Tela, F. G., et al. (2019). | Truly representative (non-random sampling) * | Justified and satisfactory. * | Comparable and the response rate is satisfactory. * | Used validated measurement tool. ** | The study controls for the most important factor. * | Self-report. * | Clearly described* | 8 |
| 13 | Terfassa, T. G., et al. (2025). | Truly representative (non-random sampling) * | Justified and satisfactory. * | Comparable and the response rate is satisfactory. * | Used validated measurement tool. ** | The study controls for the most important factor. * | Self-report. * | Clearly described* | 8 |

***Note: This tool consists of three sections; the first section evaluates the outcome and statistical analysis of each study, with a maximum of two stars; the second section, graded out of five stars, assesses the methodological quality of the study; and the third section examines the comparability of study groups or cohorts, with up to two stars possible. Studies receiving a total rating of ≥ 5 out of 10 stars were considered high quality.***
