# Supplementary material for: Magnitude and determinants of gestational weight gain in Ethiopia: a systematic review and meta-analysis
Source: Matern Health Neonatol Perinatol. 2026 Jun 10;12:23. doi: 10.1186/s40748-026-00270-x (PMC13251280; doi:10.1186/s40748-026-00270-x)
Supplement: Supplementary file 3 — Supplementary Material 3 [file 40748_2026_270_MOESM3_ESM.pdf]

Supplementary Figures S1–S7

Supplementary figure 1:

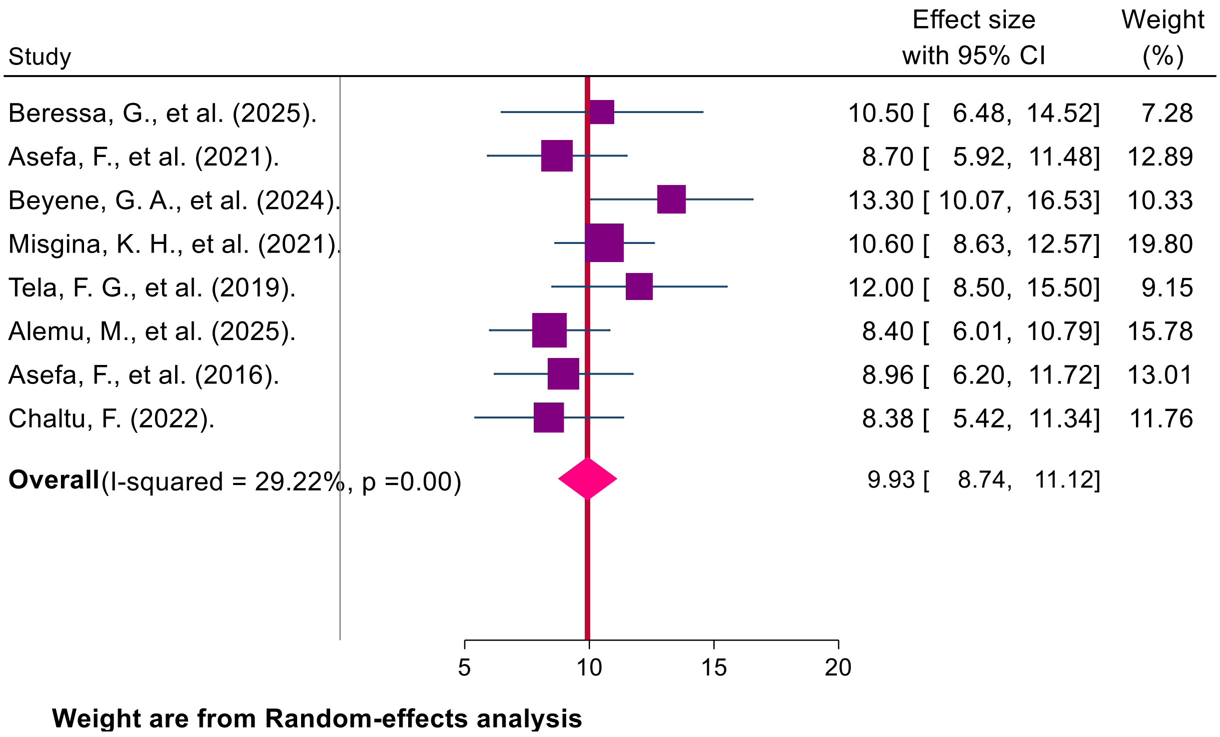

Figure 1: Pooled mean gestational weight gain among pregnant women in Ethiopia.

Supplementary figure 2:

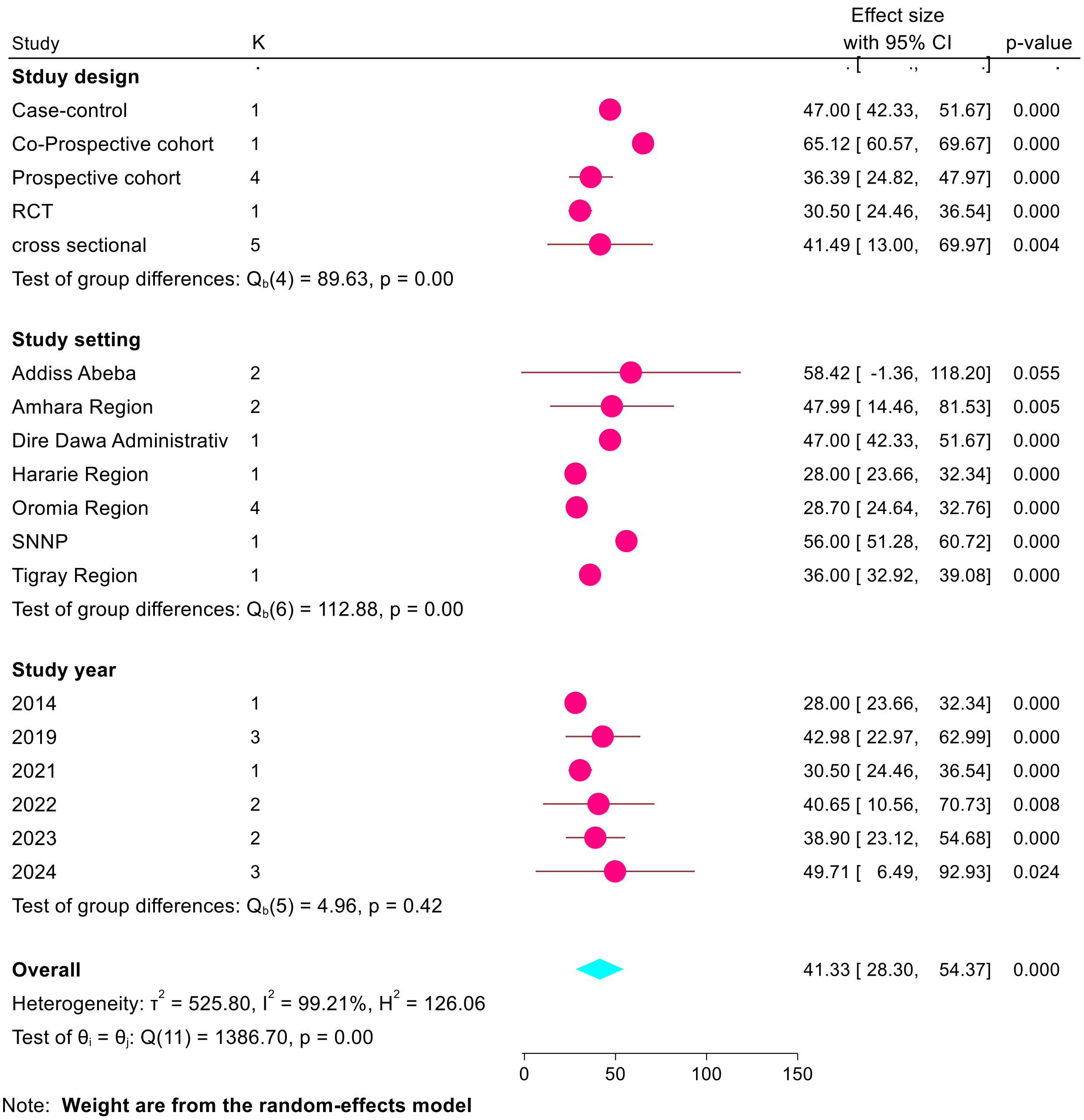

Figure 2: Subgroup analysis for adequate gestational weight gain.

Supplementary figure 3:

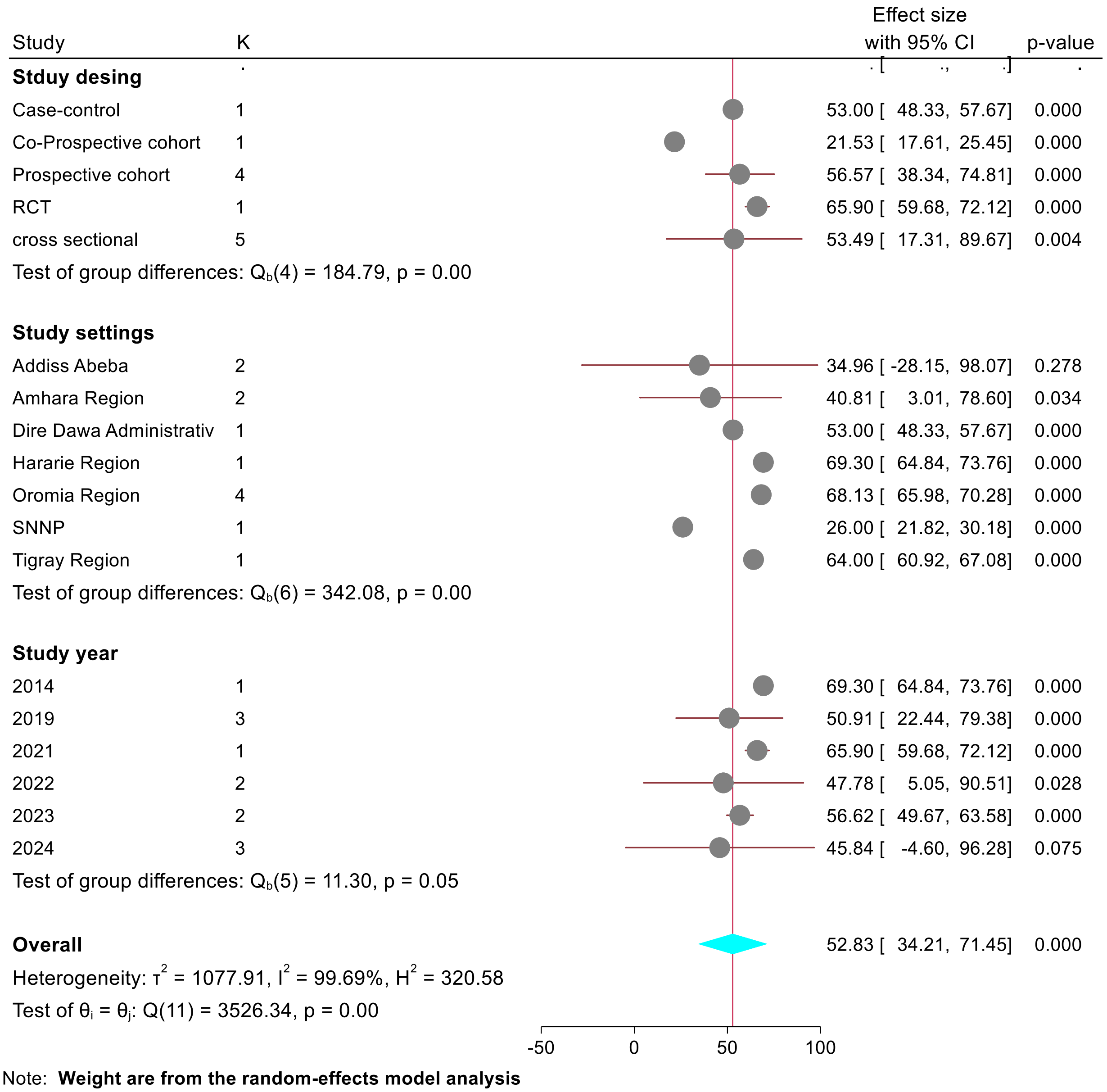

Figure 3: Subgroup analysis for inadequate gestational weight gain.

Supplementary figure 4:

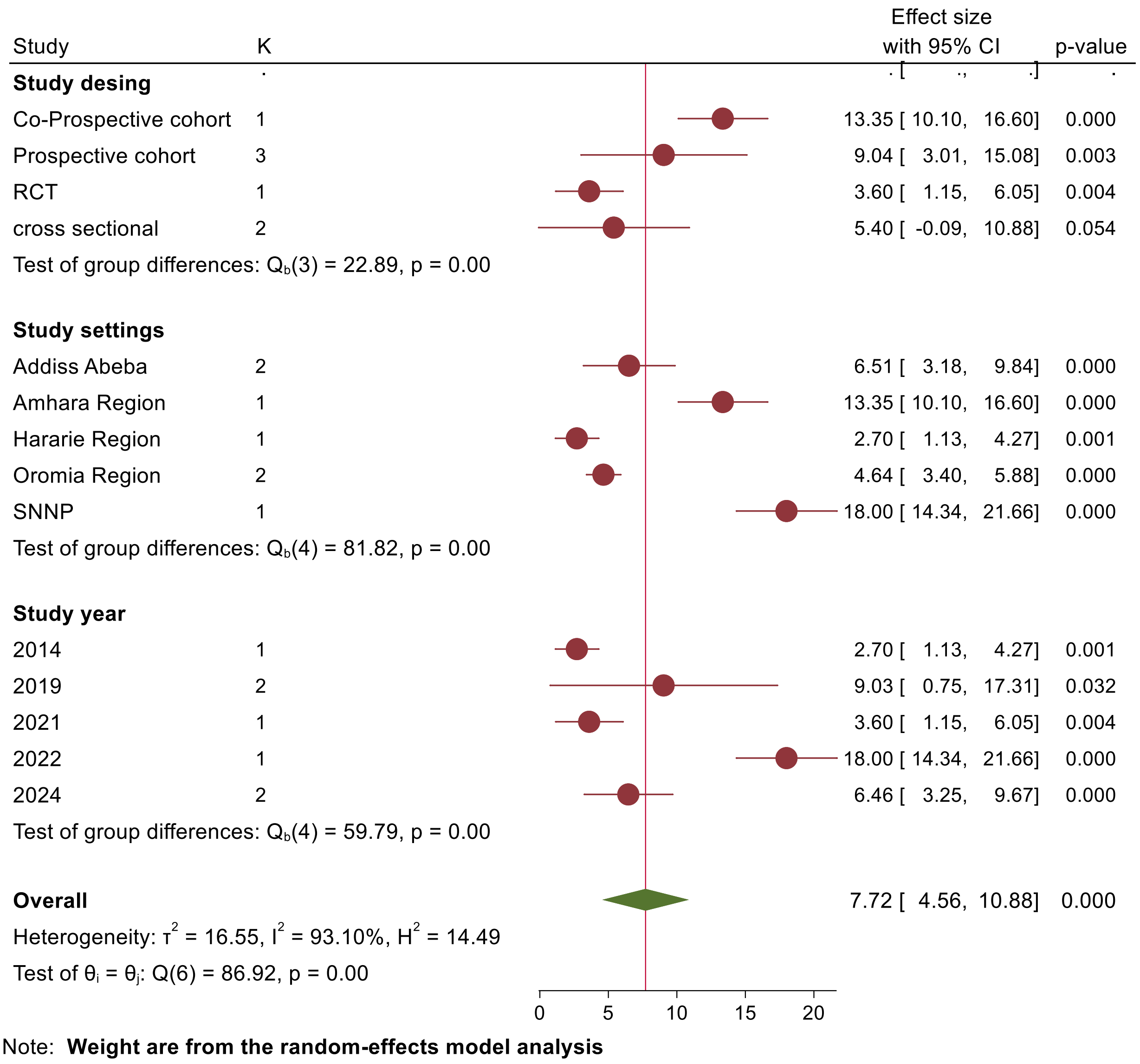

Figure 4: Subgroup analysis for excessive adequate gestational weight gain.

Supplementary figure 5:

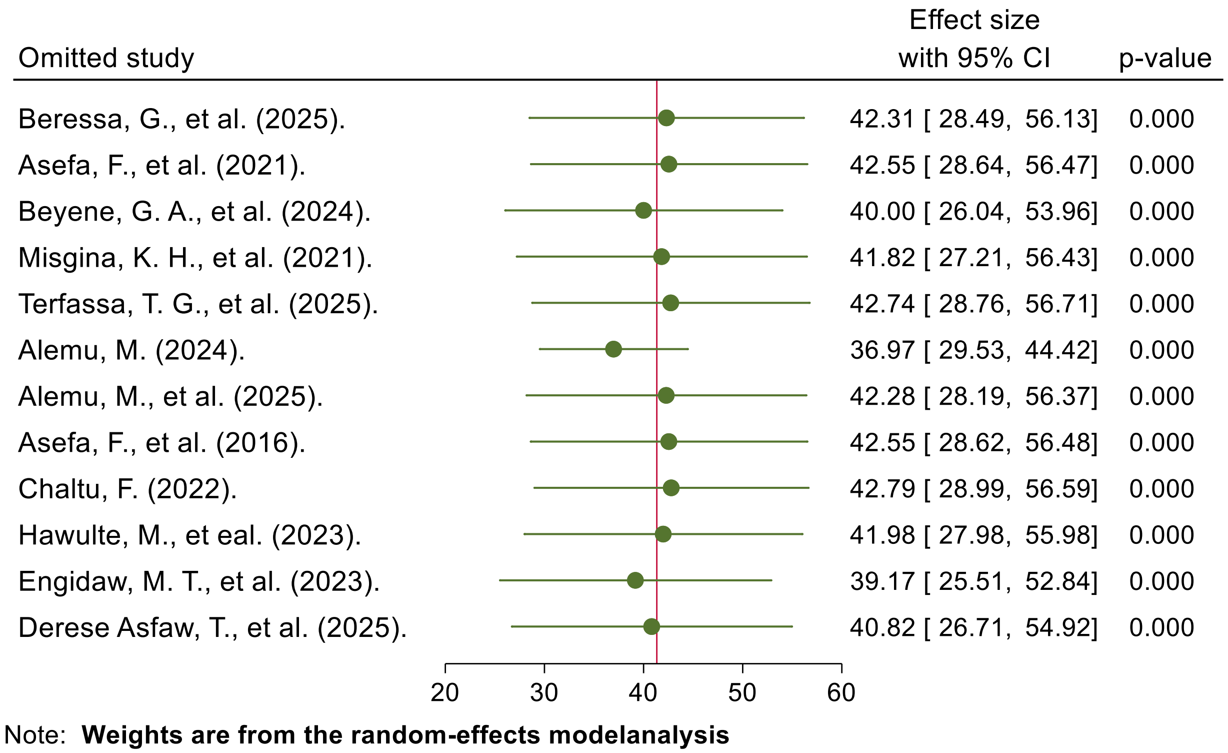

Figure 5: Leave-one-out meta-analysis for adequate gestational weight gain.

Supplementary figure 6:

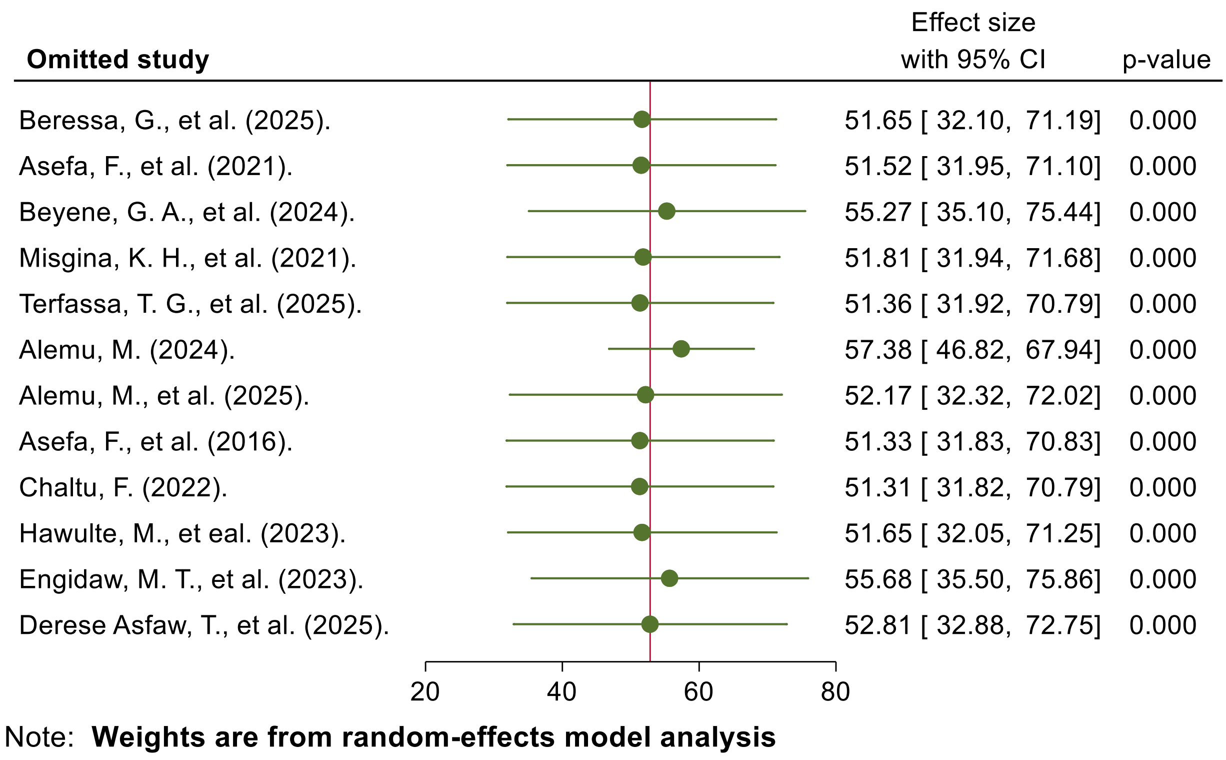

Figure 6: Leave-one-out meta-analysis for inadequate gestational weight gain.

Supplementary figure 7:

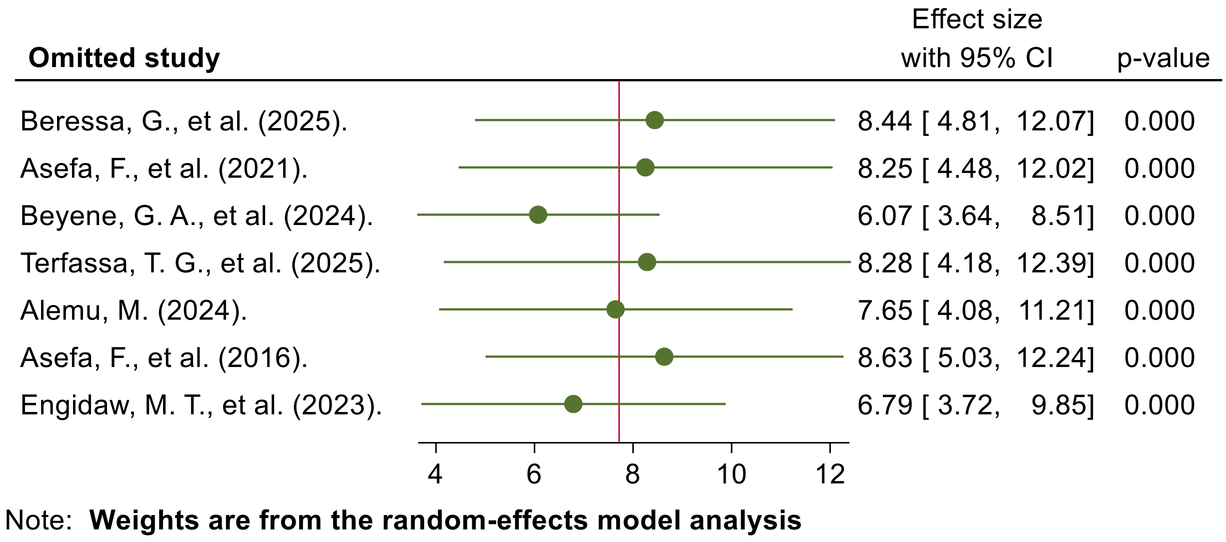

Figure 7: Leave-one-out meta-analysis for excessive gestational weight gain.
