# Supplementary material for: Magnitude and determinants of gestational weight gain in Ethiopia: a systematic review and meta-analysis
Source: Matern Health Neonatol Perinatol. 2026 Jun 10;12:23. doi: 10.1186/s40748-026-00270-x (PMC13251280; doi:10.1186/s40748-026-00270-x)
Supplement: Supplementary file 4 — Supplementary Material 4 [file 40748_2026_270_MOESM4_ESM.pdf]

Supplementary Figures S8–S12 for determinants of gestational weight gain.

Figure 8

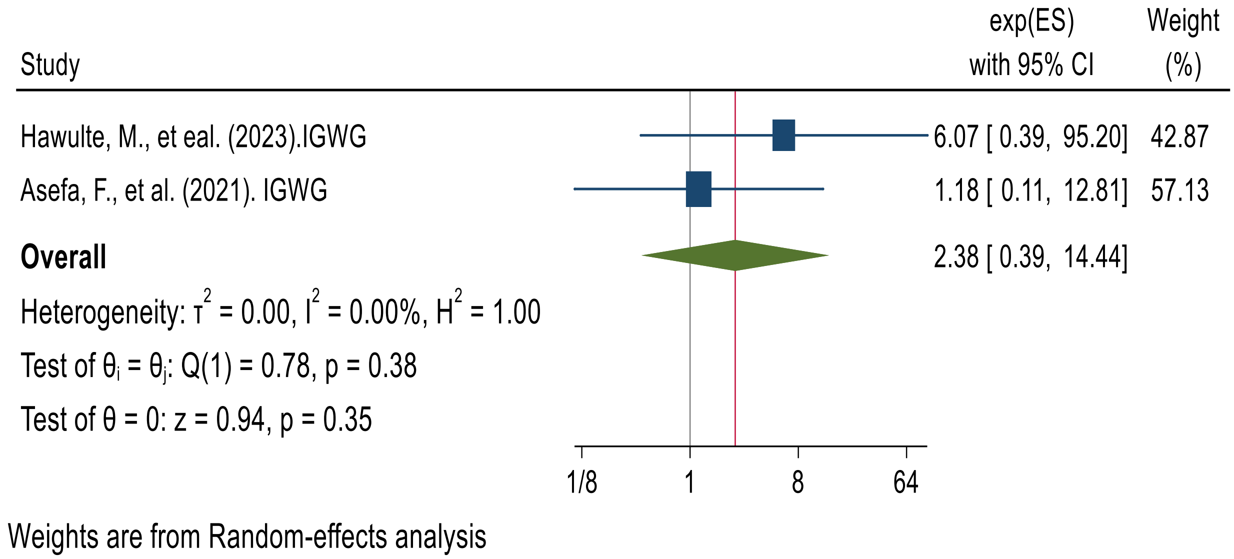

Figure 1: Forest plot showing the association between low dietary diversity (<5 food groups) and inadequate gestational weight gain.

Figure 9

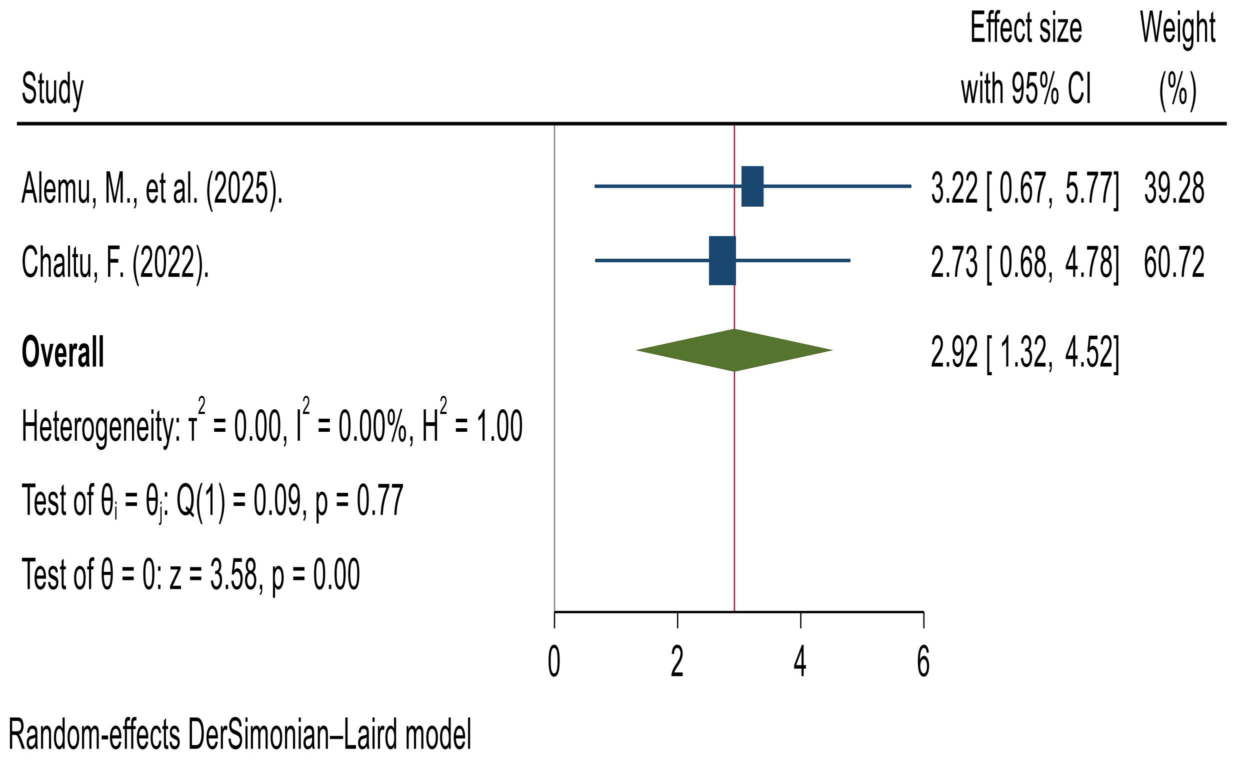

Figure 2: Forest plot showing the association between Meal frequency >3 times /day and adequate gestational weight gain.

Figure 10

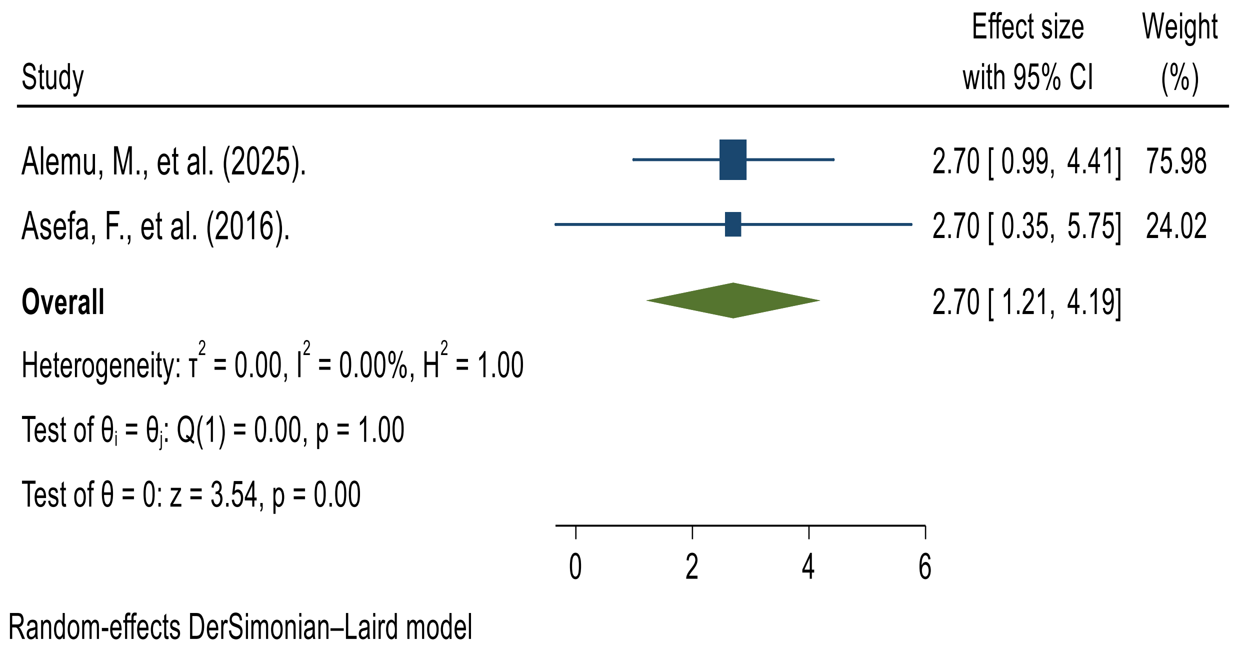

Figure 3: Forest plot showing the association between Daily animal-source food consumption and adequate gestational weight gain.

Figure 11

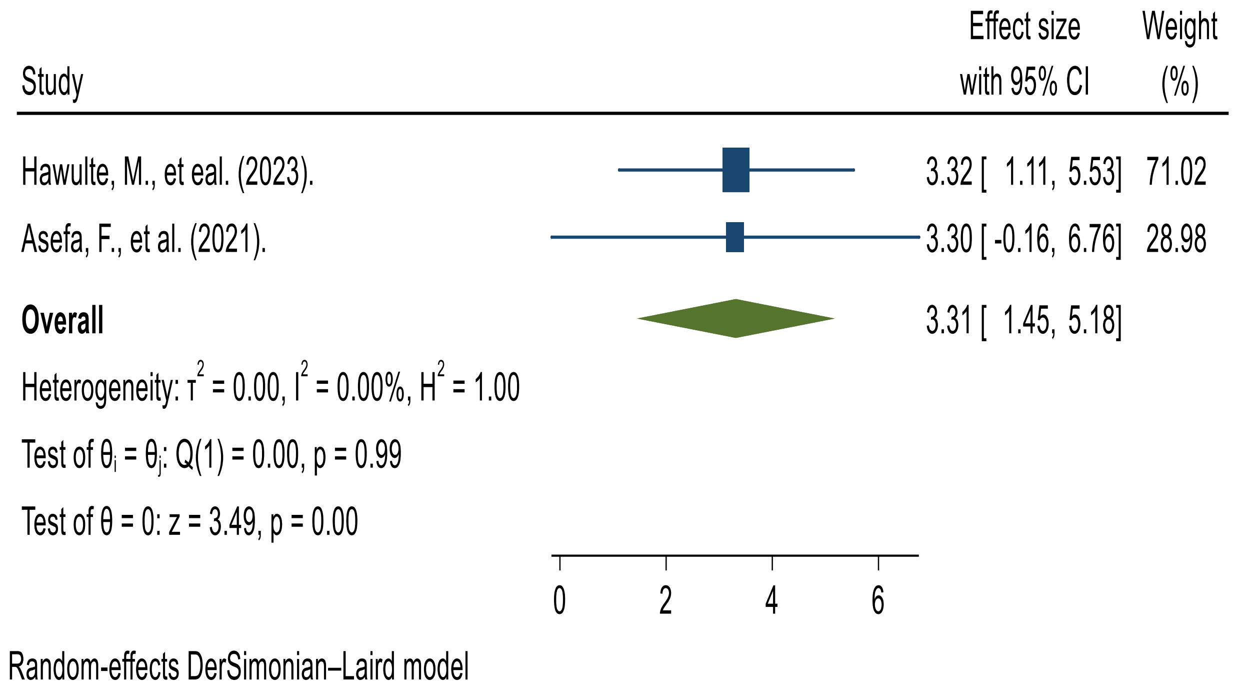

Figure 4: Forest plot showing the association between early-pregnancy underweight with inadequate gestational weight gain.

Figure 12

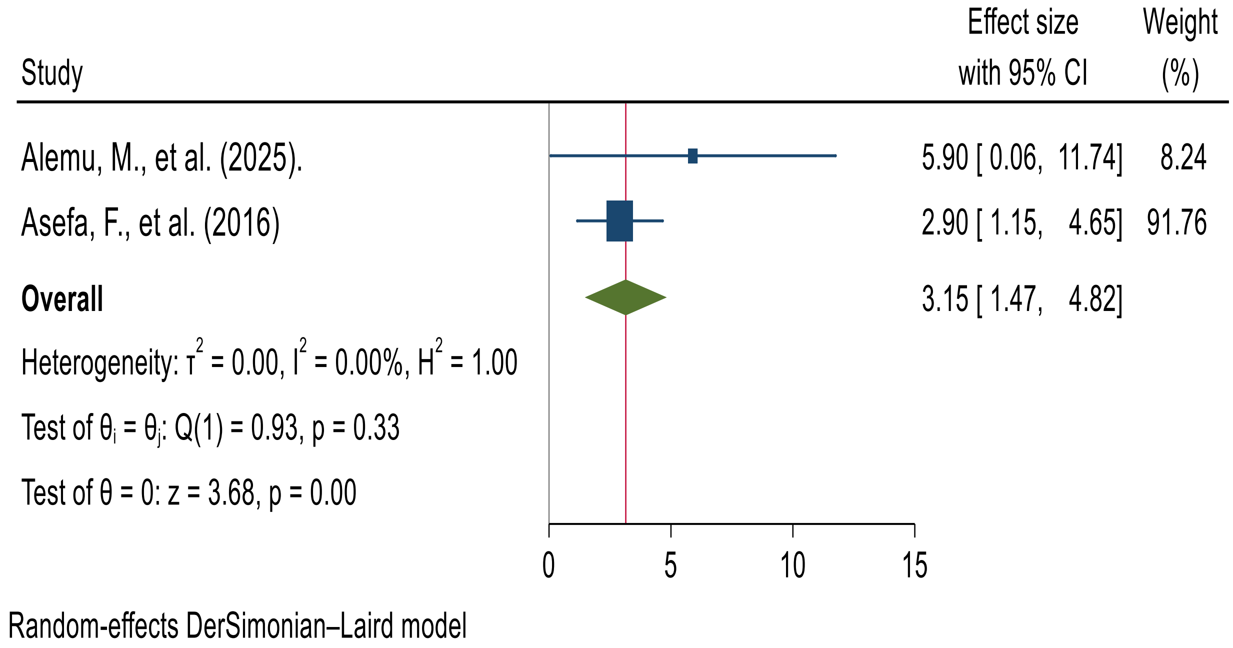

Figure 5: Forest plot showing the association between ANC visits >3 with adequate gestational weight gain.
